# Hemoperitoneum in a patient with spontaneous rupture of the posterior wall of an unscarred uterus in the second trimester of pregnancy

**DOI:** 10.1186/s13104-015-1575-0

**Published:** 2015-10-24

**Authors:** Nabil Abdalla, Malgorzata Reinholz-Jaskolska, Michal Bachanek, Krzysztof Cendrowski, Ryszard Stanczak, Wlodzimierz Sawicki

**Affiliations:** Department of Obstetrics, Gynecology and Oncology, Second Faculty of Medicine, Medical University of Warsaw, Kondratowicza Street 8, 03-242 Warsaw, Poland; Department of Obstetrics and Gynecology, District Hospital in Wolomin, Wolomin, Poland

**Keywords:** Caesarean section, Hemoperitoneum, Hysterectomy, Pregnancy, Uterine rupture

## Abstract

**Background:**

Hemoperitoneum resulting from a rupture of an unscarred uterus is a rare condition. Uterine rupture in patients without evident risk factors is associated with non-specific signs and symptoms that can delay the diagnosis. This is a report of spontaneous rupture of posterior wall of the uterus in the second trimester of pregnancy presented as intra-abdominal bleeding.

**Case presentation:**

Here, we report the case of a 31-year-old Caucasian multiparous female (gravida 3, para 1) who had a sudden onset of abdominal pain at 28 weeks of gestation. The patient had no history of caesarean section. Exploratory laparotomy was performed due to deterioration of the patient’s clinical condition, and ultrasound results were suspicious for hemoperitoneum. Uterine rupture in the posterior wall with active bleeding from the defect was confirmed. A caesarean section was performed, and a live female infant weighing 1000 g, with an Apgar score of three, was delivered. A hysterectomy was performed during the caesarean section.

**Conclusion:**

Diagnostic difficulties arise from the rarity of the disease, a nonspecific clinical picture and the absence of the main risk factors. Uterine rupture should be considered in the differential diagnosis of hemoperitoneum in patients with an unscarred uterus.

## Background

There are no precise diagnostic criteria of uterine rupture during pregnancy and labor. Due to the different clinical presentations of uterine rupture, many cases (e.g., asymptomatic uterine rupture) have been excluded from population-based studies [1]. According to a systematic review of maternal morbidity and mortality by the World Health Organization in 2005, the median incidence of uterine rupture is 5.3 per 10,000 deliveries. A history of caesarean section is the most important risk factor for uterine rupture in developed countries [1]. Most reported cases of uterine rupture are associated with previous scarring of the uterus, multiparity, a short length of time (less than 18 months) since the last caesarean section, the number of previous caesarean sections, single-layer closure instead of two-layer closure, placenta previa and the use of prostaglandins or oxytocin for labor induction or augmentation [2–5]. Fetal heart rate abnormality, most commonly bradycardia, is the most common presentation of uterine rupture. Uterine rupture can also present as abdominal pain, vaginal bleeding, and altered uterine contractions. More rarely, it can present as hypotension, shock, hematuria, shoulder tip pain and scar tenderness. The most common combination of these symptoms is an abnormal fetal heart rate with abdominal pain. Uterine rupture can be an accidental finding during a caesarean section [3]. Maternal tachycardia is an alarming sign that can, along with another medical signs, alert the physician to the possibility of uterine rupture [6]. It was thought that loss of uterine contraction is a typical sign of uterine rupture; however, normal uterine contraction patterns or hyperstimulation of the uterus may also be noted [7]. In the differential diagnosis of uterine rupture, placental abruption, placenta previa, uterine inversion, cervical tear, vaginal tear, coagulopathy, uterine atony and uterine artery rupture may be considered [8]. Intra-abdominal bleeding is rare after the first trimester of pregnancy. In the first trimester of pregnancy, most cases of intra-abdominal bleeding are related to extrauterine pregnancy [9]. Hemoperitoneum in the second trimester can be attributed to both obstetric and non-obstetric causes. A literature review revealed few reports of cases of hemoperitoneum due to obstetric causes other than ectopic pregnancy [10]. The causes of these cases can be divided into placental, uterine and vascular causes. Placenta percreta is a rare placental abnormality that can cause severe complications, such as hemoperitoneum [11]. Placental abruption is not a cause of hemoperitoneum in the absence of uterine rupture. However, during pregnancy, the clinical features of hemoperitoneum can trigger a suspicion of placental abruption because these conditions share similar clinical features, and these similarities can cause diagnostic difficulties [12]. Uterine anomalies are a reported cause of rupture of the unscarred uterus in the first trimester in patients with uterine anomalies [13]. Endometriosis can cause erosion of the utero-ovarian vessels, resulting in severe hemorrhage [14].

### Case presentation

**Fig. 1:**
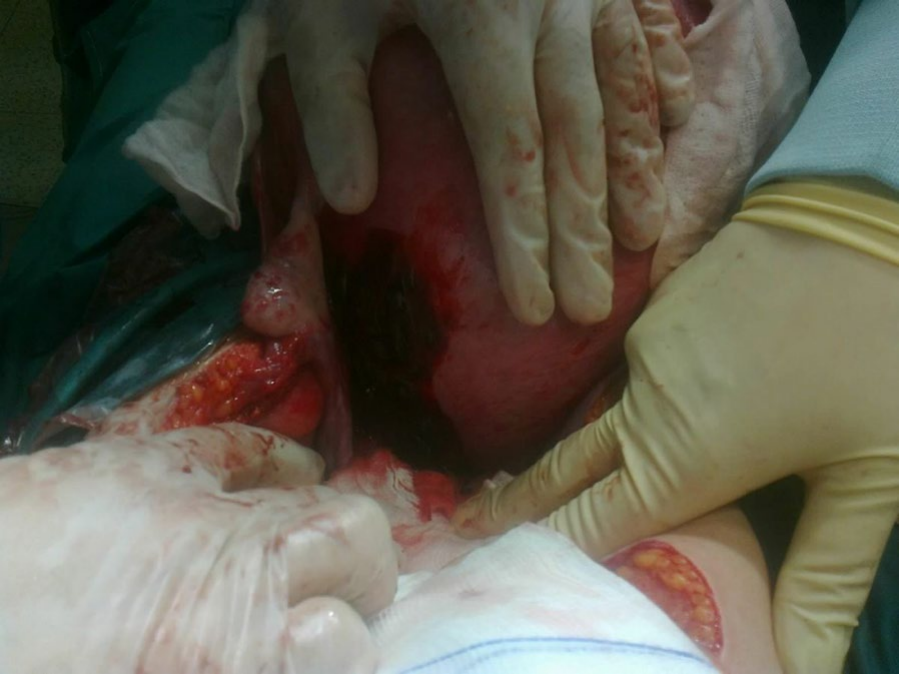
The macroscopic appearance of the uterus during laparotomy at the time of diagnosis. The left side of the posterior wall is ruptured, with active bleeding. The left ovary and left fallopian tube are intact

A 31-year-old pregnant Caucasian female, gravida 3, para 1, at 28 weeks of gestation was admitted to the hospital complaining of a sudden onset of moderate lower abdominal pain. She denied trauma. The patient’s past medical history was unremarkable. There was no history of intrauterine device. The patient reported one uncomplicated miscarriage, followed by uterine curettage, after which she became pregnant and delivered vaginally at term. Uterine perforation was not suspected during uterine curettage, on the other hand no laparoscopy procedure was done after curettage to exclude injury. The second pregnancy and delivery were uneventful. The course of the recent pregnancy had also been uneventful. A physical examination revealed vital signs that were within normal ranges. An abdominal examination revealed that her abdomen was soft and tender. The uterus was soft and not tender, and contractions were not present. The patient was feeling fetal movements well, and a fetal non-stress test showed that the fetus was reactive. The severity of pain increased gradually. Initial laboratory investigations showed no abnormalities. An ultrasound scan showed a live fetus in a longitudinal lie with cephalic presentation. The placenta was on the anterior wall of the uterus. Abnormal placental localization and hematoma were not found. The amniotic fluid index was in the normal range. The pain was increasing gradually, and the next day, the patient was transported to a tertiary referral hospital. Upon admission, the patient’s abdomen was tender, with guarding. Her vital signs were within normal range. The uterus was soft and not tender, with irregular uterine contractions. The patient was feeling fetal movements well, and the result of a fetal non-stress test was reactive. An ultrasound scan was performed again, and it revealed free fluid inside the peritoneal cavity. In the Pouch of Douglas, there was a 75 × 35 mm nonvascularized area, which suggested the presence of a blood clot. The other abdominal structures were without abnormalities. A blood analysis showed a 2 g/dL decrease in hemoglobin level. An exploratory laparotomy was performed, and 1000 mL of blood was evacuated from the abdominal cavity. The adnexa were without macroscopical changes. The left parametrium was enlarged and purplish. The fundal height was appropriate for the number of weeks of pregnancy, without any macroscopic changes in the anterior wall. The enlarged gravid uterus was moved gently forward to reveal the posterior wall. On the posterior wall, there was a defect (approximately 10 cm) in the muscular wall, with active bleeding (Fig. 1). The amniotic sac was intact inside the uterus. No pelvic adhesions were found, and no endometriosis foci were found. Bleeding was excluded from the gastrointestinal tract, spleen, liver, kidneys and the mesentery. Due to the extent of the defect and the magnitude of the bleeding, a caesarean section was performed, followed by hysterectomy. A live female infant weighing 1000 g, with Apgar scores of three was delivered. The estimated blood loss during surgery was 2000 mL. Six units of packed red cells and three units of fresh frozen plasma were given to the patient. Histopathological examination of the uterus did not reveal muscle disease or endometriosis. The patient was discharged from the hospital in good general condition after 11 days. The baby was discharged from the hospital after 70 days, also in good general condition.

## Conclusion

In this case, no risk factor for uterine rupture was evident. There might be unrecognized uterine perforation during curettage and there was no diagnostic laparoscopy after this procedure to exclude this injury. Uterine curettage have been reported as a cause of uterine rupture during next pregnancy [15]. Previous injury of the uterus is not likely to have caused uterine rupture in this case because the second pregnancy was uneventful. On the other hand this possible mild unrecognized injury might be further worsened by the second pregnancy and labor. For this reason possible unrecognized uterine injury should be considered as a cause of the uterine rupture in this case. A careful obstetric history did not suggest the presence of endometriosis. Moreover, no evidence of endometriosis was found during the laparotomy, and adenomyosis was excluded by histopathological examination of the excised uterus. In this case, the placenta was located in the anterior abdominal wall; thus, a relationship between the placenta and the defect can easily be excluded [11]. The patient is generally healthy, and her family history is negative for muscle diseases. A rupture of unscarred uterus is rare [16]. The ultrasound exam was beneficial in this case because it revealed the presence of free fluid inside the abdominal cavity. The results of the fetal non-stress test were reactive, which led us initially to exclude uterine rupture in the differential diagnosis and delayed the decision to perform an emergency caesarean section. An emergency exploratory laparotomy was performed to determine the site of bleeding, which could have been obstetric or non-obstetric. Difficulties in the diagnosis of uterine rupture can be attributed to several factors. First, uterine rupture is rare. A scarred uterus is the main risk factor for uterine rupture. In patients with atypical histories, the diagnosis may be delayed or may even be established at the time of laparotomy, thereby increasing maternal and fetal morbidity and mortality. Rupture of the uterus should be considered in pregnant women with hemoperitoneum, even when caesarean section is absent from the obstetric history.

## Consent

Written, informed consent was obtained from the patient for publication of this Case Report and accompanying images.

## References

[1] Hofmeyr GJ, Say L, Gülmezoglu AM (2005). WHO systematic review of maternal mortality and morbidity: the prevalence of uterine rupture. BJOG.

[2] Veena P, Habeebullah S, Chaturvedula L (2012). A review of 93 cases of ruptured uterus over a period of 2 years in a tertiary care hospital in South India. J Obstet Gynaecol.

[3] Fitzpatrick KE, Kurinczuk JJ, Alfirevic Z (2012). Uterine rupture by intended mode of delivery in the UK: a national case-control study. PLoS Med.

[4] Bujold E, Goyet M, Marcoux S (2010). The role of uterine closure in the risk of uterine rupture. Obstet Gynecol.

[5] Hagneré P, Denoual I, Souissi A, Deswarte S (2011). Spontaneous uterine rupture after myomectomy. Case report and review of the literature. J Gynecol Obstet Biol Reprod.

[6] Sweeten KM, Graves WK, Athanassiou A (1995). Spontaneous rupture of the unscarred uterus. Am J Obstet Gynecol.

[7] Rodriguez MH, Masaki DI, Phelan JP, Diaz FG (1989). Uterine rupture: are intrauterine pressure catheters useful in the diagnosis?. Am J Obstet Gynecol.

[8] Mazzone ME, Woolever J (2006). Uterine rupture in a patient with an unscarred uterus: a case study. WMJ.

[9] Agdi M, Tulandi T (2009). Surgical treatment of ectopic pregnancy. Best Pract Res Clin Obstet Gynaecol.

[10] Salama S, Nizard J, Camus E, Ville Y (2009). Spontaneous haemoperitoneum after the second trimester of pregnancy. Diagnosis and management. Eur J Obstet Gynecol Reprod Biol.

[11] Blé RK, Adjoussou S, Doukoure B (2011). Placenta percreta: a rare etiology of spontaneous uterine perforation in the second trimester of pregnancy. Gynecol Obstet Fertil.

[12] Vyjayanthi S, Rajesh U, Bloomfield TH (2002). Haemoperitoneum due to placenta percreta in the third trimester mimicking placental abruption. J Obstet Gynaecol.

[13] Tola EN (2014). First trimester spontaneous uterine rupture in a young woman with uterine anomaly. Case Rep Obstet Gynecol.

[14] Williamson H, Indusekhar R, Clark A, Hassan IM (2011). Spontaneous severe haemoperitoneum in the third trimester leading to intrauterine death: case report. Case Rep Obstet Gynecol.

[15] Nkwabong E, Kouam L, Takang W (2007). Spontaneous uterine rupture during pregnancy: case repoert and review of literature. Afr J Reprod Health.

[16] Sun HD, Su WH, Chang WH, Wen L, Huang BS, Wang PH (2012). Rupture of a pregnant unscarred uterus in an early secondary trimester: a case report and brief review. J Obstet Gynaecol Res.

